# Diabetic Striatopathy: A Case Report of a Patient With Poor Glycaemic Control and Abnormal Movements

**DOI:** 10.7759/cureus.45581

**Published:** 2023-09-19

**Authors:** Kasun E Chithrapathra, Waruni S Hewanayake, Samitha Egodage, Shehan Silva

**Affiliations:** 1 Internal Medicine, Colombo South Teaching Hospital, Kalubowila, LKA; 2 Radiology, Army Hospital, Colombo, LKA; 3 Medicine, University of Sri Jayawardenapura, Gangodawila, LKA

**Keywords:** gemistocytes, hemiballismus-hemichorea, hyperglycaemia, hyperkinetic movement disorder, diabetic striatopathy

## Abstract

Diabetic striatopathy (DS) is a condition occurring in individuals with type II diabetes mellitus (T2DM) where there are abnormal (usually single-sided) bodily movements (hemiballismus-hemichorea (HBHC)). DS involves the interaction between diabetes leading to damage to areas such as the striatum with the development of a noticeable hyperkinetic movement disorder. Here, we present a case of a 72-year-old man with T2DM, ischaemic heart disease, and dyslipidaemia, who presented with involuntary movements of the bilateral upper limbs (the left side more affected than the right) for three weeks along with progressively worsening subtle involuntary movements of the mouth and tongue, with intact speech, swallowing, and gait. The neurological examination revealed high-amplitude intermittent, sudden onset involuntary movements of the bilateral upper limbs, primarily affecting the left side. Based on clinical findings, which were supported by imaging studies, a diagnosis of diabetic striatopathy was made. His presentation was beyond the classical presentation of unilateral involvement seen in HBHC, but with early identification and strict glycemic control, satisfactory improvement of his clinical status was achieved.

## Introduction

Diabetic striatopathy (DS) is an uncommon hyperkinetic movement disorder arising from nonketotic or hyperketotic hyperglycaemia. The overall prevalence of this condition is documented as one in 100,000 population worldwide; however, it is believed that the actual prevalence may be higher, primarily due to the lack of awareness and missed diagnoses [1]. This condition is commonly documented to occur in elderly females with type II diabetes mellitus (T2DM) and is associated with hemiballismus-hemichorea (HBHC) [1]. It is diagnosed clinically with the support of various imaging modalities including computed tomography (CT), magnetic resonance imaging (MRI), etc. T1 sequence of MRI of the basal ganglia will show hyperintensities, especially involving the contralateral striatum [2]. The main management modality of DS is strict glycaemic control, yet in patients in whom the chorea is severe, anti-chorea medications like dopamine antagonists (haloperidol), or gamma-aminobutyric acid (GABA) agonists (clonazepam) can be initiated in the early course of the disease. 

## Case presentation

A 72-year-old man with T2DM, ischaemic heart disease, and dyslipidaemia presented with involuntary movements of the bilateral upper limbs (the left side more affected than the right) for three weeks. Additionally, there were some subtle involuntary movements of the mouth and tongue. The symptoms had progressively worsened. However, the patient’s speech, swallowing, and gait were normal.

He had no previous history of trauma, no history of similar symptoms, and there was no family history of movement disorders. His medications included aspirin, atorvastatin, enalapril, bisoprolol, metformin, gliclazide, and sitagliptin in therapeutic doses. The patient had exhibited poor drug compliance for the past month. There was no significant occupational or environmental exposure to toxins, the patient consumed alcohol only during social gatherings, and he was an occasional smoker.

On admission, he was afebrile and well hydrated with a blood pressure of 130/80mmHg, and normal cardiac, respiratory, and abdominal examination. The neurological examination revealed high-amplitude involuntary, intermittent, sudden onset, violent, flinging movements of the bilateral upper limbs, primarily affecting the left side. There were also subtle flickers of movements of the tongue and lips. The patient's gait and other neurological functions including the cerebellar system were normal. Kayser-Fleischer rings were not detected. There were no stigmata of connective tissue disease or metabolic or nutritional disorders.

**Figure 1:**
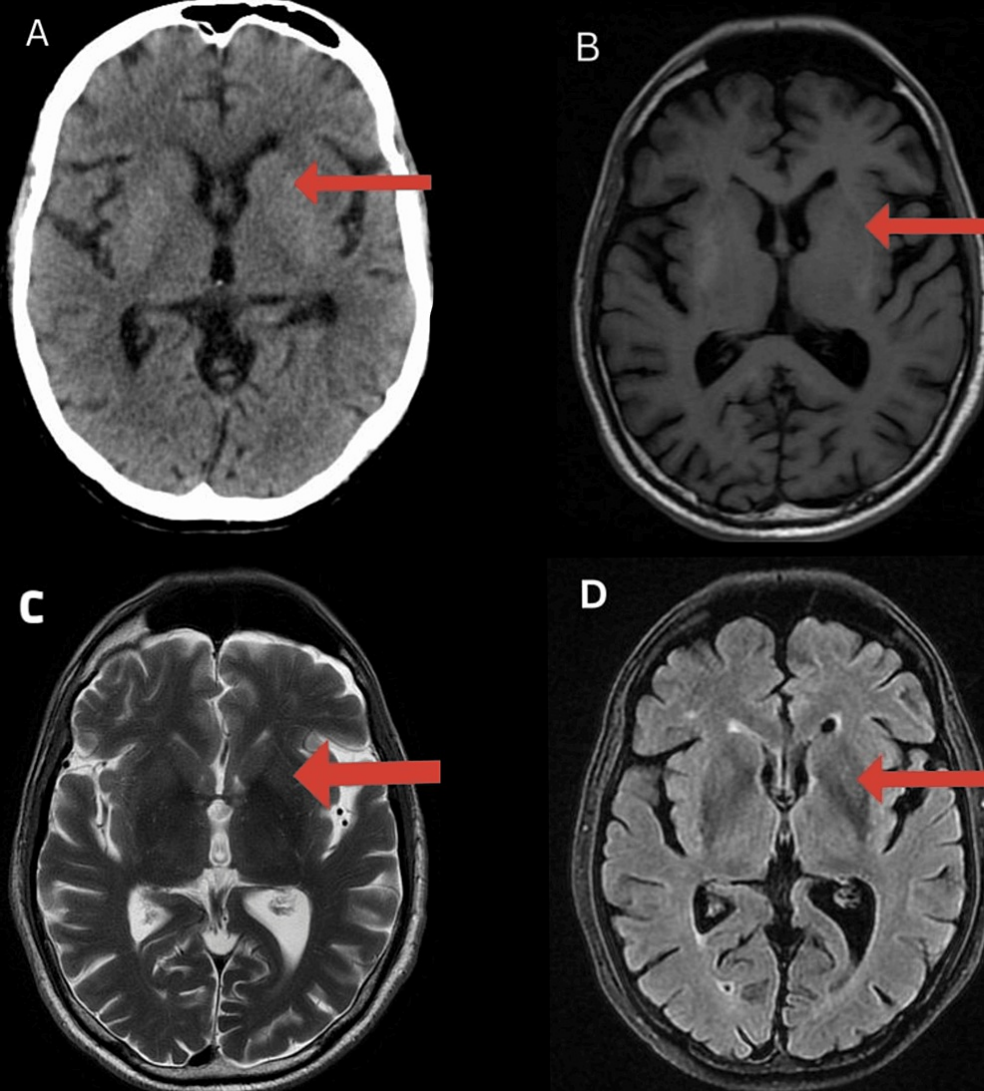
(A) Non-contrast CT scan of the brain; (B) MRI T1 sequence; (C) MRI T2 sequence; (D) MRI FLAIR sequence Arrowed areas show changes in contralateral striatum (putamen and caudate nuclei), possibly due to diabetic striatopathy. FLAIR: fluid-attenuated inversion recovery

On admission, his glycated haemoglobin (HbA1C) level was 8.5%. A non-contrast CT scan of the brain revealed bilateral hyperdensities in the basal ganglia (Figure 1a). Furthermore, an MRI of the brain showed heterogenous hyperintensities in T1 sequences (Figure 1b) with hypointensities in T2 and fluid-attenuated inversion recovery (FLAIR) sequences (Figures 1c-1d) in the bilateral putamen and caudate nuclei. This was suggestive of striatopathy.

Based on the clinical, biochemical and radiological findings, a probable diagnosis of diabetic striatopathy was made and the patient was initiated on treatment. His glycaemic control was achieved with subcutaneous insulin for which a significant clinical improvement was observed in 12 hours. Investigations to rule out other potential differential diagnoses such as rheumatic fever, hereditary haemochromatosis, Wilson disease, and other metabolic imbalances were done (Table 1). There was no evidence of infection. Furthermore, the absence of dehydration and urinary ketone bodies excluded diabetic ketoacidosis.

**Table 1:** Investigations

Investigations		Value	Reference Range
Full Blood Count	White Blood Cells (× 10^9^ /L)	11	4.5 – 11
	Haemoglobin (g/dL)	11.4	11-13
	Platelets (x 10^9^/L)	245	150 – 400
Liver Biochemistry	Aspartate Transaminase (U/L)	38	< 40
	Alanine Transaminase (U/L)	26	< 40
	Serum Albumin (g/L)	38	35 - 50
	Alkaline phosphatase (U/L)	102	< 120
	Gamma glutamyl transferase (U/L)	96	5 - 40
	INR (International Normalizing Ratio)	0.98	<1
Renal Profile	Serum Creatinine (mg/dl)	158	20 - 275
	Blood Urea (mg/dl)	14	6 - 24
Electrolytes	Serum Sodium (mmol/L)	139	135 – 145
	Serum Potassium (mmol/L)	4.6	3.5 – 5.5
	Serum Calcium (mmol/L)	2.5	2.2 to 2.7
	Serum Magnesium (mg/dl)	1.02	1.6 – 2.5
Venous Blood Gas Analysis	pH	7.4	7.35 – 7.45
	Serum Bicarbonate (mEq/L)	20	22 - 26
Thyroid Function Test	Thyroid Stimulating Hormone ( mIU/L)	0.5	0.5 to 5.0
Other Investigations	Serum Ferritin (ng/ml)	108	24 - 336
	Serum Ceruloplasmin (mg/dl)	20	14 - 20
	Serum Anti Streptolysin O Titer (IU/ml)	76	<200

After the achievement of glycaemic control, the patient was transitioned to twice-a-day premixed insulin. By the third day, he had a full resolution of all involuntary movements. The patient was reviewed as an outpatient every two weeks and was found not to have abnormal neurological findings in the setting of well-controlled fasting and postprandial glycaemia. 

## Discussion

The defining characteristic of DS is the emergence of HBHC, accompanied by increased signal intensity observed on T1-weighted MRI scans in specific regions of the brain, including the putamen (commonly), caudate nucleus, and globus pallidus. Similar findings can be elicited in other imaging modalities as well. The internal capsule is usually unaffected. This condition usually occurring in unilateral presentation can manifest very rarely bilaterally [2,3], as in our patient. Different combinations of involvement are observed [4]. Typically, imaging studies reveal the absence of any mass effect or contrast enhancement, indicating that the blood-brain barrier remains intact in DS [5]. The underlying pathology of DS is postulated to involve the destruction of myelin, caused by swollen reactive astrocytes known as gemistocytes [6]. DS has a higher incidence in females with advancing age as the most significant risk factor [7].

The presence of acute HBHC serves as a distinctive clinical feature of DS. However, diagnosing DS can be challenging due to the existence of several other conditions with similar HBHC symptoms [8]. HBHC can have various underlying causes, which encompass metabolic, infectious, inflammatory, iatrogenic, trauma, autoimmune, and neurogenetic/neurodegenerative factors [8]. Although there is a wider range of potential causes for HBHC, a thorough evaluation of the clinical presentation and relevant laboratory findings can aid in narrowing down the differential diagnosis to a select number of potential causes. In the case of DS, typical clinical features include HBHC affecting one side of the body, a characteristic pattern of progression from the upper to lower limbs, exacerbation of HBHC during periods of nervousness, and a tendency for HBHC to diminish during sleep. [3] By examining the underlying causes of T1 hyperintensities observed in the striatum on brain MRI scans, the list of potential differential diagnoses can be further refined and narrowed down. Based on the principles of T1-weighted MRI sequences, T1-shortening resulting in hyperintensities can be caused by various factors, including melanin, subacute haemorrhage with methaemoglobin, fat, slow-velocity blood flow, high protein content, and the presence of paramagnetic transition metals such as manganese, iron, zinc, and copper, which have unpaired electrons [9]. The clinical features observed in DS can aid in the exclusion of certain causes of T1 hyperintensity. For example, disorders related to copper deposition (i.e. Wilson disease) and neuroferritinopathy such as neurodegeneration associated with pantothenate kinase, typically present as chronic diseases with a gradual onset [10].

Effective management of this hyperkinetic movement disorder often involves maintaining optimal glucose control. In some cases, treatment with medications that reduce abnormal movements, such as dopamine antagonists (e.g., haloperidol), vesicular monoamine uptake inhibitors (e.g. tetrabenazine), or gamma-aminobutyric acid agonists (e.g., clonazepam), may be necessary [3].

## Conclusions

DS is an uncommon hyperkinetic movement disorder linked to uncontrolled diabetes and distinguished by HBHC. Bilateral manifestation of the disease is a rare occurrence, hence the uniqueness of this case. Diagnosing DS can be challenging due to the various potential causes of HBHC. Employing a dual strategy that considers the patient's clinical symptoms and the specific characteristics observed in brain MRI can serve as an effective diagnostic tool for DS. Future research efforts should aim to establish a standardized protocol for the diagnosis of DS.
